# Criteria for symptom remission revisited: a study of patients affected by schizophrenia and schizoaffective disorders

**DOI:** 10.1186/1471-244X-13-235

**Published:** 2013-09-26

**Authors:** Federica Pinna, Massimo Tusconi, Marta Bosia, Roberto Cavallaro, Bernardo Carpiniello

**Affiliations:** 1 Department of Public Health, Clinical and Molecular Medicine-Section of Psychiatry, University of Cagliari, Via Liguria 13, 09127 Cagliari Italy; 2 Department of Clinical Neurosciences, San Raffaele Scientific Institute, Via Stamira d’Ancona 20, 20127 Milan, Italy; 3 Institute for Advanced Study, IUSS, Center for Neurolinguistics and Theoretical Syntax (NeTS), Pavia, Italy

## Abstract

**Background:**

This study aims to compare severity criteria defined by the Remission in Schizophrenia Working Group (RSWGcr) with other criteria in relation to functional and neurocognitive outcome.

**Methods:**

112 chronic psychotic outpatients were examined. Symptomatic remission according to RSWGcr was compared with the outcome achieved using criteria based on PANSS Positive and Negative Scales (PANSS-PNScr) and the entire PANSS (PANNS-TScr).

**Results:**

Remission rates were 50%, 35% and 23% respectively at RSWGcr, PANSS-PNScr and PANNS-TScr; functional remission rates were 32%, 42% and 54%. Sensitivity, specificity, predictive value and ROC analysis demonstrated the superiority of PANSS-PNScr in identifying patients with higher functional and cognitive outcomes. Regression analysis showed a significant predictive effect of PANSS-TScr on functioning. General linear model analyses demonstrated significantly higher mean scores at PSP and BACS for patients remitted according to PANSS-TScr.

**Conclusion:**

The use of more restrictive severity criteria of remission seems to be associated with improved identification of truly remitted patients.

## Background

It is widely acknowledged that recovery may be achieved even in subjects affected by serious mental illnesses. However, the dimensions to be included in the concept of recovery are still the object of ongoing debate [1]. It is generally assumed that recovery will comprise both objective and subjective [2] components, otherwise defined as clinical and personal domains [3]; the objective component generally refers to clinical outcomes which are evaluated by means of operationally defined criteria; subjective recovery refers to the ongoing process of positive changes in an individual’s subjective experience of themselves as human beings [1]. Clinical objective and personal subjective recovery are largely independent phenomena [4] and both should be considered as targets for therapeutic interventions in schizophrenia and related disorders. Symptom remission represents the fundamental component underlying clinical recovery, together with improved functioning [1], and is viewed as the main target for psychopharmacological interventions [5]. Up until fairly recently, a univocal method for the assessment of remission was lacking; to this aim, a significant step forward was represented by publication of the Remission in Schizophrenia Working Group criteria (RSWGcr) [6] which proved to be conceptually viable and easy to use both in clinical trials and clinical practice [7].

According to RSWGcr [6] clinical remission is based on a symptom severity criterion comprising eight items of the PANSS scale chosen as being the most diagnostic-specific for schizophrenia, and a duration criterion, thus excluding symptom domains not diagnostically relevant for the disorder. As PANSS scale provides ratings investigating not only symptom severity per se but also functional impairment, a score of “mild” or better (i.e. 3 points or less) at all eight “core” symptoms was considered sufficiently representative of a level of impairment consistent with symptomatic remission of the disorder [7]. According to recent reviews [8,9] reported remission rates vary widely across studies (17-88%), likely due to use of symptom severity criterion alone in the majority of studies [9]. A number of studies have demonstrated the validity of these remission criteria using two different approaches, namely comparison of different definitions of symptomatic remission and association of remission criteria with various outcome dimensions, mainly overall symptomatic status and functional outcome [10]. However, several recent studies seem to highlight the potential limitations of severity criteria as currently conceived in predicting functioning and other important outcome variables. Indeed, a study aimed at investigating symptomatically remitted and non remitted patients demonstrated a significantly better level of functioning for remitted patients, although the latter continued to display significant areas of inadequate functioning, low levels of subjective wellbeing and moderate-severe emotional distress [11]. Moreover, a recent study attempting to provide an ecological validation for the symptomatic remission criterion, showed how although remitted patients reported fewer positive symptoms, better mood states and partial recovery of reward experience, remission status was not related to functional recovery [12]. Starting from these premises, and taking into account the need for further investigation into the validity of current criteria for symptomatic remission, the present study was devised to compare the efficacy of three different and increasingly “stringent” sets of criteria in evaluating remission by means of PANNS in relation to functional and cognitive status.

## Methods

### Sample

In the context of an ongoing study on recovery [13], all outpatients with a diagnosis of schizophrenia or schizoaffective disorder according to DSM-IV-TR attending a university community mental health centre (CMHC) in the year 2010 were enrolled consecutively. Patients with other comorbid psychiatric and or somatic disorders were also included in the study, with the exception of those with comorbid mental retardation or organic brain diseases. Standard care was provided to patients as in CMHCs in Italy (clinical monitoring at least on a monthly basis; pharmacological treatment; home care when required, psychosocial and rehabilitation interventions tailored to patient’s needs). The study was approved by the institutional Ethical Committee of Local Health Unit of Cagliari (Italy) and was conducted according to national laws.

### Ratings

Evaluation was performed by residents in psychiatry using a set of standardized methods of evaluation, after adequate training in use of all instruments adopted. Personal and social data, and clinical history were collected through a structured interview purpose-developed for the study. After providing informed consent, patients were interviewed by means of the Italian versions [14,15] of SCID-I [16] and SCID-II [17]; inter-rater reliability, assessed using Cohen’s K before the study, was higher than 0.80. Symptom severity was evaluated using the Italian version [18] of PANSS (Positive and Negative Syndrome Scale) [19]; as previously, interviews were conducted by residents in psychiatry trained in use of the instrument using the Italian version [20] of SCI-PANSS (Structured Clinical Interview for the positive and Negative scale) [21]; ratings were based on criteria indicated in the PANSS Manual [22]; inter-rater reliability of PANSS evaluations in terms of ICC (Intraclass correlation coefficient) for the PANSS total score ranged from 0.65 to 0.95. Wherever possible, PANSS assessment included a standard section of queries addressed to treating clinicians and to caregivers. RSWG criteria [6] based on ratings at 8 focal symptoms in positive, negative and general psychopathology subscales of PANSS (P1, P2, P3, N1, N4, N6, G5, G9) were applied for clinical remission; patients were judged to be in clinical remission according to a severity criterion (scores obtained at each of these items had to be ≤ 3 points, indicating mild severity of symptoms). Due to the cross-sectional nature of the study, clinical remission was evaluated taking into account the severity criterion alone, excluding the duration criterion (remission maintained for six-months). Moreover, two more restrictive severity criteria for remission were adopted: obtaining scores ≤3 at each item of Positive and Negative (PANSS-PNScr) or of Positive, Negative and General Psychopathology Scale of PANSS (PANSS-TScr). The reason for this choice is that the scope of the study was to test the performance of PANSS in evaluating symptom remission, as this scale is generally used in common clinical practice; as a consequence, we decided to use the three subscales originally identified by the developers of the instrument, in spite of the fact that several factor analyses have underlined how a five-factor model better characterizes PANSS data [23].

Overall clinical status was also evaluated by the Clinical Global Impression-Schizophrenia scale (CGI-SCH) [24]. Cognitive functioning was evaluated by means of the Brief Assessment of Cognition in Schizophrenia scale (BACS) [25], using five of the six subtests, namely list learning, digit sequencing, category instances and controlled oral word association test, symbol coding and executive functions; a gender/age/education adjusted score and thus an equivalent score were calculated [26]. Mini Mental State Examination test (MMSE) [27] was administered, calculating an age/education adjusted score [28]. Functioning was evaluated by PSP (Personal and Social Performance Scale) [29], which assesses social functioning of patients in 4 main areas: socially useful activities, personal and social relationships, self-care and disturbing/aggressive behaviours. A non-standardized interview was conducted with the patient, caregivers (when available) and the treating physician, with the aim of assessing functioning by means of PSP. A comprehensive overall score ranging from 1 (maximum dysfunction) to 100 (maximum functioning) was attributed, based on score obtained at each single area. A total score exceeding 70 indicates a condition of “functional remission”, with scores being related to overall good functioning.

### Statistical analysis

Categorical data were analyzed using Pearson’s *χ*2 Test or Fisher’s exact test; continuous variables were assessed by means of Student’s”t” test for independent samples. The magnitude of differences in mean scores obtained at different rating scales used in the study was calculated by means of Cohen’s “d”. To evaluate differences in remission rates observed according to the different proposed criteria, McNemar Test for matched pairs of subjects was used. ROC analysis, with Sensitivity, Specificity and Predictive Value Analysis of the ability of different definitions of Remission to predict good outcomes were calculated. Moreover, multiple linear regressions and General Linear Model Analysis were employed to investigate whether the proposed definitions of clinical remission would predict functional and cognitive outcomes. Data analyses were performed using SPSS 19.0. Level of significance was set at a p value ≤ 0.05 for two-tailed hypothesis.

## Results

### Baseline characteristics

The sample comprised 112 patients: 80 males (71.4%) and 32 (28.6%) females, forty-six (41.1%) schizophrenic and sixty-six (58.9%) schizoaffective subjects (58.9%); mean age was 43.5 +/− 9.42 years (range 25–68); mean years of education 10.84+/−3.9 (range 4–24); 97 subjects (86.6%) were single; 83 (74.1%) unemployed.

### Clinical remission

To evaluate differences in remission rates, McNemar Test for matched pairs of subjects was used. The proportion of remitted patients was significantly higher using RSWGcr (50%), compared to both PANSS- PNScr (34.8%) and PANSS-TScr (23.2%) (p < .0001 and p < .0000 respectively); the frequency of remission was significantly (p < .0008) higher with PANSS-PNScr respect to PANSS-TScr. Remitted and non-remitted patients featured several significantly different characteristics as shown in Table 1. A higher proportion of remitted patients, increasing in line with stringency of criteria adopted, was found among schizoaffective subjects, although differences were generally not statistically significant. Mean scores at CGI-SCH were all significantly higher among non-remitted patients yielding effects of medium (Cohen’s d 0.5-1) or large (Cohen’s d > 1) magnitude for the majority of scales, independent of remission criteria (Table 2). Significantly higher mean scores were also found among non-remitted patients at PANSS, with effects of large magnitude, independent of remission criteria (Table 2). Significantly different mean scores were detected for cognitive functioning (Table 3), using both PANSS-PNScr and PANSS-TScr, between remitters and non remitters at MMSE and at almost all subtests of BACS, with effects of medium magnitude, independent of criteria adopted.

**Table 1:** Sociodemographic characteristics of remitted and non-remitted patients according to different criteria

**Items**	**Criteria of remission**	**Remitted**	**Non-remitted**	**Statistics (df)**
Education (years)	SWGC*	11.55 (4.16)	10.13 (3.43)	t(110) = 1.981, p = .05
(Means ± SD)	PANSS PNS**	12.59 (4.09)	9.90 (3.42)	t(110) = 3.697, p < .0001
	PANSS TS***	13.38 (4.13)	10.07 (3.45)	t(110) = 4.094, p < .0001
Occupation (unemployed) N (%)	SWGC*	36 (64.3%)	47 (83.9%)	Chisq(1) = 9.775, p < .0001
	PANSS PNS**	22 (56.4%)	61 (83.6%)	Chsq(1) = 8.402, P = .004
	PANSS TS***	14 (53.8%)	69 (80.2%)	ChiSq(1) = 21.694, P < .0001
Course of illness (continuous + episodic with residual symptoms) N (%)	SWGC*	39 (69.6%)	50 (89.3%)	Chisq(1) = 9.560, p = .008
	PANSS PNS**	25 (64.1%)	64 (87.8%)	Chisq(1) = 9.879, p = .007
	PANSS TS***	15 (57.7%)	74 (86.1%)	Chisq(1) = 12.611, p = .002
Duration of illness (months)	SWGC*	163.68 (100.01)	227.48 (112.58)	t(110) = −3.171, p = .002
(Means ± SD)	PANSS PNS**	138.10 (87.88)	226.29 (109.93)	t(110) = −4.323, p < .0001
	PANSS TS***	134.58(102.30)	214.02(017.03)	t(110) = −3.350, p = .001

*SWGC Schizophrenia Working Group Severity Criterion.

**PANSSPNS PANSS Positive and Negative Scores Severity Criterion.

***PANSSTS PANSS TOTAL Score Severity criterion.

**Table 2:** Mean scores ± sd at clinical scales of remitted and non-remitted patients according to different criteria

**Items**	**Criteria of remission**	**Remitted**	**Non-remitted**	**Statistics (df)/Cohen’s d**
CGI-S positive symptoms	SWGC*	1.60 (0.95)	2.95 (1.42)	t(110) = −5.853, p < .0001/1.17
	PANSS PNS**	1.47 (0.89)	2.79 (1,41)	t(110) = 4.860, p < .0001/1.04
	PANSS TS***	1.27 (0.67)	2.59 (1.39)	t(110) = 4.678,p < .0001/1.209
CGI-S negative symptoms	SWGC*	1.78 (0.91)	3.36 (1.27)	t(110) = 7.478,p < .0001/1.43
	PANSS PNS**	1.68 (0.93)	3.04 (1.32)	t(110) = −5.650, p < .0001/1.19
	PANSS TS***	1.54 (0.71)	2.92 (1.36)	t(110) = 4.948,p < .0001/1.20
CGI-S depressive symptoms	SWGC*	1.71 (0.85)	2.36 (1.31)	t(110) = 3.076,p = .003/0.58
	PANSS PNS**	1.70 (0.90)	2.21 (1.24)	t(110) = −2.185 p = .03/0.46
	PANSS TS***	1.38 (0.64)	2.29 (1.30)	t(110) = 3.423p = .001/0.88
CGI-S cognitive symptoms	SWGC*	1.84 (1.03)	3.18 (1.20)	t(110) = 6.298,p < .0001/1.19
	PANSS PNS**	1.66 (0.91)	2.96 (1.26)	t(110) = 5.631,p < .0001/1.18
	PANSS TS***	1.65 (0.98)	2.78 (1.28)	t(110) = 4.134p < .0001/0.99
CGI-S overall severity	SWGC*	2.45 (0.95)	3.82 (0.76)	t(110) = 8.309,p < .0001/1.59
	PANSS PNS**	2.34 (0.96)	3.56 (0.92)	t(110) = 6.479p < .0001/1.29
	PANSS TS***	2,12 (0.82)	3.49 (1.01)	t(110) = 6.305,p < .0001/1.49
PANSS positive scale	SWGC*	8.96 (2.09)	14.39 (4.35)	t(110) = 8.417,p < .0001/1.59
	PANSS PNS**	8.51 (1.71)	13.37 (4.40)	t(110) = 6.617p < .0001/1.45
	PANSS TS***	8.19 (1.77)	12.73 (4.36)	t(110) = 5.168p < .0001/1.36
PANSS negative scale	SWGC*	10.57 (3.65)	18.70 (5.85)	t(110) = 8.803,p < .0001/1.66
	PANSS PNS**	9.64 (2.91)	17.30 (6.07)	t(110) = 7.420,p < .0001/1.61
	PANSS TS***	9.58 (3.03)	16.16 (6.30)	t(110) = 5.139,p < .0001/1.33
PANSS general psychopathology	SWGC*	21.98 (4.87)	32.68 (7.48)	t(110) = 8.964,p < .0001/1.69
	PANSS PNS**	22.13 (5.13)	30.11 (8.31)	t(110) = 5.462,p < .0001/1.15
	PANSS TS***	19.92 (3.58)	29.57 (7.98)	t(110) = 5.972,p < .0001/1.66
PANSS Total scale	SWGC*	41.52 (7.92)	65.77 (13.87)	t(110) = 11.354,p < .0001/1.69
	PANSS PNS**	40.28 (7.85)	60.78 (15.58)	t(110) = 7.697,p < .0001/1.66
	PANSS TS***	37.69 (6.61)	58.47 (15.65)	t(110) = 6.578,p < .0001/1.75

*SWGC Schizophrenia Working Group Severity Criterion.

**PANSSPNS PANSS Positive and Negative Scores Severity Criterion.

***PANSSTS PANSS TOTAL Score Severity Criterion.

**Table 3:** Mean score ± sd at Neuropsychological Tests in remitted and non-remitted patients according to different criteria

**Items**	**Criteria of remission**	**Remitted**	**Non-remitted**	**Statistics (df)/Cohen’s d**
MMSE Total score	SWGC*	26.80 (2.71)	25.02 (4.42)	t(110) = 2.576,p = .011/0.485
	PANSS PNS**	27.33 (2.61)	25.15 (4.06)	t(110) = 3.033,p = .003/0.638
	PANSS TS***	27.62 (2.47)	25.40 (3.93)	t(110) = 2.713,p = .008/0.676
BACS list learning	SWGC*	9.31 (4.77)	9.92 (4.59)	t(99) = −.652,p = 0.516/NA°
	PANSS PNS**	9.10 (5.24)	9.89 (4.35)	t(99) = −.806,p = 0.422/NA°
	PANSS TS***	8.62 (5.42)	9.94 (4.38)	t(99) = 1.236,p = 0.219/NA°
BACS Digit sequencing task	SWGC*	14.75 (6.06)	11.26 (6.19)	t(99) = 2.86,p = .005/0.571
	PANSS PNS**	16.30 (5.65)	11.23 (6.01)	t(99) = 4.112p < .0001/0.969
	PANSS TS***	17.31 (5.47)	11.56 (5.98)	t(99) = 4.247p < .0001/1.00
BACS Verbal Fluency/category istances	SWGC*	9.89 (4.98)	8.10 (4.96)	t(99) = 1.801,p = .075/0.360
	PANSS PNS**	10.66 (4.27)	8.10 (5.20)	t(99) = 2.479,p = .014/0.538
	PANSS TS***	10.75 (4.21)	8.40 (5.16)	t(99) = 2.056,p = .042/0.499
BACS Verbal FluencyControlled Oral Words ass.test	SWGC*	15.05 (3.89)	14.50 (5.49)	t(99) = 0.569 p = .571/NA°
	PANSS PNS**	15.46 (4.31)	14.41 (4.96)	t(99) = 1.061 p = .27/NA°
	PANSS TS***	15.21 (4.89)	14.63 (4.73)	t(99) = 0.536 p = .593/NA°
BACS Symbol coding	SWGC*	32.60 (13.64)	28.60 (12.29)	t(99) = 1.549,p = 0.125/NA°
	PANSS PNS**	36.10 (12.87)	27.67 (12.29)	t(99) = 3.228,p = .002/0.669
	PANSS TS***	37.85 (12.48)	28.20 (12.43)	t(99) = 3.263,p = .001/0.774
BACS Tower of London	SWGC*	11.38 (5.98)	9.5 (6.53)	t(99) = 1.445,p = 0.152/NA°
	PANSS PNS**	12.80 (5.44)	9.25 (6.33)	t(99) = 2.815,p = .006/0.601
	PANSS TS***	13.16 (5.65)	9.60 (6.21)	t (99) = 2.536,p = .013/0.599

*SWGC Schizophrenia Working Group Severity Criterion.

**PANSSPNS PANSS Positive and Negative Scores Severity Criterion.

***PANSSTS PANSS TOTAL Score Severity Criterion.

°*NA* not assessed in absence of significant difference.

### Functioning

Twenty-three patients (20.5%) were found to be in functional remission. The rates of “functionally remitted” patients detected were invariably and significantly higher among clinically remitted patients (Table 4); functional remission increased from 32.1% among patients in clinical remission according to RSWGcr, to 42.1% among remitted subjects according to PANSS-PNScr, reaching 53.8% among the remitted according to PANSS-TScr. Rates of patients reaching a score < 3 (substantial absence of impairment) at each PSP subscale were significantly higher among clinical remitters, independent of the criterion adopted; similarly, an increasing proportion of patients showing no impairment was detected with more stringent criteria (PANNS-PNScr and PANSS-TScr). Mean scores at PSP subscales were all significantly higher among non-remitters, indicating poorer functioning, independent of criteria; even mean PSP total score was significantly lower among non-remitters, independent of remission criteria. Magnitude of effect sizes increased on a par with stringency of clinical remission adopted, at least with regard to PSP socially useful activities, social relationships and total scale.

**Table 4:** Results at PSP scale in remitted and non-remitted patients according to different criteria

**Items**	**Criteria of Remission**	**Remitted**	**Non-remitted**	**Statistics(df)/Cohen’s d**
PSP –activities (Means ± SD)	SWGC*	1.88 (1.27)	3.20 (1.21)	t(110) = 5.642,p < .0001/−1.06
	PANSS PNS**	1.51 (1.21)	3.08 (1.18)	t(110) = 6.660,p < .0001/−1.31
	PANSS TS***	1.15 (1.05)	2.95 (1.22)	t(110) = 6.814,p < .0001/−1.58
PSP-social rel (Means ± SD)	SWGC*	2.02 (1.15)	2.86 (1.15)	t(110) = 5.642,p < .0001/−0.73
	PANSS PNS**	1.80 (21.14)	2.73 (1.17)	t(110) = 3.600,p < .001/-0.81
	PANSS TS***	1.65 (1.02)	2.67 (1.18)	t(110) = 3.975,p < .0001/-0.92
PSP -self care (Means ± SD)	SWGC*	0.34 (0.69)	0.80 (1.16)	t(110) = 2.559,p < .012/-0.48
	PANSS PNS**	0.26 (0.55)	0.74 (1.12)	t(110) = 2.537,p = .013/−0.54
	PANSS TS***	0.31 (0.62)	0.65 (1.06)	t(110) = 2.062,p = .043/−0.39
PSP -aggressive and disturbing behaviour (Means ± SD)	SWGC*	0.14 (0.44)	0.50 (0.81)	t(110) = 2.896,p < .005/−0.55
	PANSS PNS**	0.10 (0.38)	0.44 (0.764)	t(110) = 2.575,p = .011/−0.56
	PANSS TS***	0.08 (0.27)	0.40 (0.40)	t(110) = 2.145,p = .034/-057
PSP Total Score (Means ± SD)	SWGC*	62.27 (13.65)	50.38 (14.79)	t(110) = 4.419,p < .0001/0.83
	PANSS PNS**	65.38 (13.13)	51.48 (14.34)	t(110) = 5.032,p < .0001/1.00
	PANSS TS***	69.77 (8.63)	52.26 (14.67)	t(110) = 5.783,p < .0001/1.45
PSP Total Pts with a score ≥70 (N, %)	SWGC*	18 (32.1)	5 (8.9)	*χ*2(1) = 7.879,p = .005
	PANSS PNS**	16 (42.1)	6 (8.3)	*χ*2(1) = 15.988,p < .0001
	PANSS TS***	14 (53.8)	9 (10.4)	*χ*2(1) = 20.442,p < .0001
PSP –activities Pts with score <3 (N, %)	SWGC*	26 (46.4)	5 (8.9 %)	*χ*2(1) = 17.841,p < .0001
	PANSS PNS**	23 (58.9)	13 (15.1)	*χ*2(1) = 18.907,p < .0001
	PANSS TS***	18 (69.2)	13 (15.1)	*χ*2(1) = 26.565,p < .0001
PSP –social rel Pts with score < 3 (N, %)	SWGC*	18 (32.2)	7 (12.5)	*χ*2(1) = 5.149 p = .023
	PANSS PNS**	13 (33.3)	12 (16.4)	*χ*2(1) = 3.562 p = .059
	PANSS TS***	16 (61.5)	15 (17.4)	*χ*2(1) = 17.253 p < .0001
PSP –self care Pts with score < 3 (N, %)	SWGC*	51 (91.1)	46 (82.1)	*χ*2(1) = 1.650 p = .199
	PANSS PNS**	37 (94.8)	60 (82.2)	*χ*2(1) = 3.936,p = .047
	PANSS TS***	24 (92.3)	73 (84.9)	*χ*2(1) = 0.417,p = .518
PSP -aggressive and disturbing behaviour Pts with score < 3 (N, %)	SWGC*	54 (96.4)	49 (87.5)	*χ*2(1) = 1.650 p = .165
	PANSS PNS**	3 (94.8)	73 (100.0)	*χ*2(1) = 0.111,p = .738
	PANSS TS***	26 (100.0)	77 (89.5)	*χ*2(1) = 1.712,p = .191

*SWGC Schizophrenia Working Group Severity Criterion.

**PANSSPNS PANSS Positive and Negative Scores Severity Criterion.

***PANSSTS PANSS TOTAL Score Severity Criterion.

### Prediction of functional outcome and cognitive status

To investigate whether the criteria proposed for clinical remission would reflect differences in functional outcome and cognitive status, diagnostic test evaluations were performed.

For global functioning, patients were classified as remitted/non remitted based on PSP total score. For cognitive performances, equivalent scores for each subtest of BACS were calculated, based on normative data from an Italian sample and the mean used as a measure of general cognitive ability, setting a cut-off of 1. RSWGcr, PANSS-PNScr and PANSS-TScr were compared on their ability to identify patients with better outcomes, using sensitivity, specificity and predictive value analysis. Youden’s index and the area under the receiver operating characteristic curve (AUROC) were also calculated to quantify performances of the three diagnostic criteria.

Results are shown in Table 5. Although positive predictive values are low for all definitions, as both good functional and cognitive outcomes occur at lower rates than achieving remission criteria, as expected, higher AUROC and Youden’s J were observed for the definition based on PANSS-PNScr. Multiple linear regressions were employed to assess whether the proposed definitions of clinical remission would predict outcomes. Remission status according to PANSS-TScr was used as categorical predictor and continuous measures of functioning (PSP total score) and cognition (BACS mean equivalent score) as dependent variables, with remissions according to RSWGcr and PANSS-PNScr as covariates. This type of analysis, used by Cassidy et al. [30], allows to evaluate whether RSWGcr alone or PANSS-PNScr continue to contribute to the prediction of functioning and cognitive status when PANSS-TScr remission is taken into account. Regression on functional outcome showed a significant predictive effect only for PANSS-TScr remission (p = .005, β = .36), while neither RSWGcr nor PANSS-PNScr alone were significant predictors of functioning. The explained variance for the model was moderate (R [2] = .26). The analysis on cognitive outcome showed no significant predictor effect for any of the remission criteria used. To further investigate the effect of clinical remission according to the different criteria proposed on functional and cognitive outcomes, a categorical variable defining progressive achievement of different criteria of remission was created. Patients were divided into 4 classes: not remitted, remitted only according to RSWGcr, remitted according to PANSS-PNScr but not PANSS-TScr and remitted according to PANSS-TScr. General linear model analyses were then performed with remission class as independent factor and functional and cognitive outcomes as dependent variables, respectively. The remission class showed a significant effect on PSP total score (F = 12.35, p < .000, R [2] = .26). Post-hoc analysis with Tukey HSD revealed a significant difference between patients remitted according to PANSS-TScr and both non-remitted patients (p = .0001) and patients who achieved remission only according to RSWGcr (p = .01).

**Table 5:** Sensitivity, Specificity and Predictive Value Analysis of the ability of the 3 definitions of Remission to predict good functional and cognitive outcomes

	**Remission criteria**	**Sensitivity (%)**	**Specificity (%)**	**PPV (%)**	**NPV (%)**	**AUROC**	**Youden’s J**
PSP	SwGc*	78	57	32	91	0.68	0.36
	PANSS PNS**	74	75	44	92	0.75	0.50
	PANSS TS***	61	87	54	90	0.74	0.47
BACS	SWGc*	59	53	27	81	0.56	0.12
	PANSSPNS**	55	70	35	84	0.62	0.25
	PANSS TS***	41	80	38	82	0.60	0.21

*SWGC Schizophrenia Working Group Severity Criterion.

**PANSSPNS PANSS Positive and Negative Scores Severity Criterion.

***PANSSTS PANSS TOTAL Score Severity Criterion.

A significant effect of remission class was also observed for cognitive abilities (F = 4.50, p = .005, R [2] = .13), evaluated as mean equivalent score of BACS subtests. Post-hoc analysis with Tukey HSD revealed a significant difference between patients remitted according to PANSS-TScr and both non-remitted patients (p = .035) and patients who achieved remission only according to RSWGcr (p = .035).

## Discussion

The RSWG remission criteria have previously been compared with criteria proposed by other authors [10]. Other studies have evaluated RSWGcr versus a modified version in terms of number of items included [31] or cut-off scores at each core item [32]. One study focused on the evaluation of accuracy of RSWGcr using PANSS total score as a “golden standard” [33]. Finally, another study [30] compared four definitions of remission based upon severity scores at SAPS and SANS instead of PANSS. To our knowledge, this is the first study to compare RSWGcr with their modified versions, by extending the number of items of PANSS used to evaluate remission.

In our sample 50% of subjects were in clinical remission according to RSWGcr [6], a proportion that decreased significantly by approximately one third using PANSS-PNScr, and was halved when adopting PANSS-TScr. These results are in contrast with those of van Os et al. [31] who found no substantial change in remission rates when including two PANSS items (namely, depression and suicidality) to the eight “core symptoms”. This discrepancy however may be explained considering that we adopted more stringent alternative criteria than those used by van Os et al. [31]. Our results are somewhat similar to those obtained by Beitinger et al. [32] who reanalysed data from six antipsychotic trials applying more stringent criteria with regard to cut-off scores used by RSWGcr; indeed, the frequency of remitted patients using the original RSWGcr was approximately 42% in both medium-term and long-term studies; using scores ≤ 2, remitted subjects were 16% and 13%, respectively; using a score of 1 they were respectively 3.4% and 5%. Based on these results the authors concluded that a choice of severity score ≤3 was a “realistic” choice, given that “more stringent thresholds yield remission frequencies that are not realistic”. In our study, not based on different scoring thresholds but rather on an extension of the number and type of PANSS items considered, a significant reduction of remission rates was obtained using alternative criteria, but which was not so marked as to be unrealistic. Moreover, clinical status evaluated by PANNS and CGI was invariably significantly better among remitted patients, independent of remission criteria adopted. It is noteworthy that only a few significant differences were detected in mean scores obtained at BACS between remitters and non remitters using SRWGcr; on the contrary, significant differences were detected in mean scores of almost all BACS subtests and MMSE between remitters and non-remitters using both PANSS-PNScr and PANSS-TScr, indicating a better neurocognitive functioning among patients judged as being in clinical remission according to the more selective criteria adopted in this study. This evidence seems to be of relevance, as cognitive performance is a strong predictor of functioning [34], and the best levels of functioning were found among patients considered to be remitted according to the alternative criteria of remission.

To confirm validity of the alternative criteria, these were compared with RSWGcr, particularly to assess impact produced on functioning. Approx. 20% of subjects were found to be “functionally remitted”; when functioning was evaluated on the basis of clinical remission status, a substantial increase in rates of functional remission was observed, ranging from 32.1% in patients clinically remitted according to RSWGcr, to 42.1% among patients remitted according to PANSS-PNScr, with a peak of 53.8% among patients remitted according to PANSS-TScr. Thus, by broadening the number and type of PANSS items used to evaluate remission, the ability to identify well-functioning patients was markedly improved. Confirmation of this was obtained by evaluating the proportion of patients in clinical remission who were devoid of significant impairment at each single dimension of PSP; with regard to “socially useful activities”, this proportion increased from 32% using RSGWcr to 42% using PANSS-PNScr and 53.8% using PANSS-TScr. The rates of patients devoid of impairment in “social relationships” were 46%, 58.9% and 69.2%, respectively. Furthermore, 36% of patients viewed as remitted according to RSWGcr were in employment, as were 44% of individuals remitted according to PANSS-PNScr, and 46% of remitters according to PANNS-TScr. Even when taking into consideration, as pointed out by Lambert et al. [10] that “functioning in schizophrenia…is probably influenced by other factors independent from remission status”, the results obtained are quite impressive, being achieved in the same set of patients, but with employment status clearly changing according to the way in which clinical remission is evaluated. Finally, as expected from previous studies [11,35], mean PSP scores obtained were unfailingly significantly higher among remitters than non-remitters, a finding that in our study was independent of the remission criteria, although the magnitude of differences in mean scores varied largely according to criteria adopted in evaluating clinical remission. Indeed, the effect sizes for remission evaluated by means of RSWGcr, PANNS-PNScr and PANSS-TScr were 1.06, 1.31 and 1.58 respectively for the ‘socially useful activities’ dimension, 0.73, 0.80 and 0.92 for ‘social relationships’, and 0.83, 1.01 and 1.45 for PSP total score; therefore, the use of more restrictive criteria to evaluate clinical remission is associated with a better assessment of how patients function in everyday life. Our findings also support the hypothesis that patients in remission according to more restrictive criteria display a better neurocognitive functioning, which may explain, at least in part, the improved vocational functioning of these patients [36].

In order to better investigate whether the remission criteria proposed would reflect differences in outcome we performed a series of specific analyses. Comparing RSWGcr, PANSS-PNScr and PANSS-TScr on their ability to identify patients with better functional and cognitive outcomes, the assessment of sensitivity, specificity, predictive value, and ROC analysis showed that PANSS-PNScr is characterized by the best performances. Using regression analysis, only PANSS-TScr remission is a significant predictor of functioning, while all remission criteria used in this study predicted cognitive outcome. The general linear model analysis adopted to further investigate the effect of different clinical remission criteria demonstrates a significant effect of remission class both on functioning and cognition, with patients judged as remitted according to PANNS-TScr showing significantly higher scores than those of patients, both remitted and non remitted, according to RSWGcr, but not exceeding scores of patients non-remitted according to PANSS- PNScr. Overall, these results confirm that the best prediction of functioning and, at least in part, of cognition, is achieved using remission criteria based on the use of all items of PANSS, followed by criteria based on the use of positive and negative items of the same scale.

Prior to drawing conclusions, several limitations characterizing the present study should be considered. First, the sample size of the study was rather limited; second, it focused solely on chronic outpatients who referred to the centre over a specific period, thus excluding patients who had moved away, refused to continue treatment or no longer needed continuing care. Therefore, the findings emerging from the study should be applied only to chronic patients undergoing long-term treatment. Additionally, as sample heterogeneity is considered one of the main flaws of remission studies [10], it should be taken into account how the present study included patients affected by both schizophrenia and schizoaffective disorders. Although remission rates observed were consistently, but not significantly, higher among patients with schizoaffective disorders, this should not detract from the relevance of our results, in view of the effective difference in remission rates between the two diagnostic groups independent of remission criteria adopted. The fact that the criterion of severity alone, without duration, was used in evaluating remission should be taken into account; indeed, this limitation prevented the drawing of any firm conclusions as to the validity of complete remission criteria. However, considering that remission studies generally demonstrate how use of the severity criterion alone is associated with higher remission rates [10] compared to use of both the severity and duration criteria, it is to be expected that if the time component is taken into account, the rates of remission found should be even lower. There is however no reason why that the proportional lowering of rates found in this study as the severity remission criteria became more stringent should not be confirmed, even if the time component is adopted in evaluating remission.

Lastly, in evaluating predictive factors for functioning and cognitive status, other important factors (i.e. pre-morbid IQ and premorbid functioning) which may be significantly involved, were not taken into consideration. Even in the light of these limitations however, the evidence obtained would seem to be of interest.

As expected from longitudinal studies demonstrating a clear positive correlation between severity of psychopathology and levels of impairment in psychosocial functioning [37], the present study confirmed the validity of severity remission criteria proposed by the RSWG, associated with a better symptomatologic and functional profile. The results obtained moreover lent further support to the findings of Van Os et al. [7], who reported how the use of standardized remission criteria in schizophrenia “had the potential to improve documentation of clinical status in medical records, by providing an objective measure of illness course and treatment effect that is applicable to routine clinical care”. Moreover, our data indicate that the use of all items of Negative and Positive Scales of PANNS, and particularly of the entire PANSS scale, seem to be associated with a better identification of truly “remitted” patients, at least when taking into consideration a better personal, social and cognitive functioning as expression of remission. The use of these criteria does not imply a risk of achieving unrealistic results; indeed, the adopting of more restrictive severity criteria was not associated with a drastic reduction of remission rates. However, further studies should be undertaken to evaluate the extent to which use of the six-month duration criterion, in addition to the more restrictive severity criterion adopted in this study, may elicit a decrease in remission rates, particularly as remission studies evaluated according to the criteria of Andreasen et al. generally demonstrate that the use of both severity and duration criteria results in the finding of lower remission rates compared to the use of the severity criterion alone [10]. Nonetheless, even taking into account the latter possibility, there is no reason to suggest that the conclusions of our study, and in particular performance of the different sets of PANSS-based remission criteria would not be confirmed even when taking into account the time component.

## Conclusions

In conclusion, the data obtained in this study underline the feasibility of using the entire PANSS scale to evaluate clinical remission, at least in a research context, although the authors are fully aware of the difficulties of implementing a similar method of evaluation in routine clinical settings. Indeed, in our experience, the time commitments involved in assessing remission according to SRWG criterion is approx 5–10 minutes, respect to approx. 20–25 and 30–35 minutes, respectively, using PANSS PNS and PANSS TS. Accordingly, we acknowledge that “rather than a substitute for the 30 items of PANSS, development of a concise outcome measure for remission would create a benchmark for treatment and maintenance goals in clinical research and general practice” [33].

## Competing interests

The authors declare that they have no competing interests.

## Authors’ contribution

FP participated to the design and coordination of the study, and helped to draft the manuscript, MT participated to manage the data base of the study, to the statistical analysis and helped to draft the manuscript, MB participated to the statistical analysis and helped to draft the manuscript, RC participated to the study design and to the draft of the manuscript, BC conceived the study, participated in its design and coordination and helped to draft the manuscript. All authors read and approved the final manuscript.

## Pre-publication history

The pre-publication history for this paper can be accessed here:

http://www.biomedcentral.com/1471-244X/13/235/prepub
